# Securing Smokefree Laws Covering Casinos and Bars in Louisiana via Messaging, Continuous Campaigning and Health Coalitions

**DOI:** 10.3390/ijerph19073936

**Published:** 2022-03-25

**Authors:** Tanner D. Wakefield, Stanton A. Glantz

**Affiliations:** Center for Tobacco Control Research and Education, University of California, San Francisco, CA 94143-1390, USA; tanner.wakefield@ucsf.edu

**Keywords:** smokefree, industry, regulation, advocacy, tobacco control

## Abstract

In this paper, we examine efforts by health organizations seeking comprehensive smokefree ordinances over Louisiana casinos and bars between 2010 and 2020 to determine best practices for increasing coverage. Bars and casinos remain less protected from secondhand smoke compared to other workplaces in the United States. Casino behavior is compared to the Policy Dystopia Model (PDM), a tobacco industry strategy framework. We performed a historical case study using snowball searches for news on the Access World News Database and the internet. We performed web searches using the names of key actors, organizations, and locations and interviewed nine participants. Starting in 2010, the Louisiana Campaign for Tobacco-Free Living ran ordinance campaigns supplemented by an ongoing statewide smokefree media initiative. Utilizing consistent strategies, including promoting performers as cultural emblems deserving protection, health organizations coalesced in New Orleans during 2014 and Baton Rouge in 2016 and 2017 to pursue ordinances. The coalitions secured ordinances in Louisiana’s population and tourism centers despite business resistance. Organizations obtained 30 smokefree laws across Louisiana by 2021. Casinos used PDM strategies to resist ordinances, indicating the framework may predict strategies by non-tobacco entities resisting tobacco control. Louisiana shows that ongoing local campaigns, social justice themes and cultural messaging with coalitions in cities can secure smokefree laws covering casinos and bars and that local ordinance campaigns are a viable method for advancing smokefree protections over those venues in states where the state legislatures are resistant to action.

## 1. Introduction

As of January 2022, casinos remain less protected by smokefree laws than other workplaces despite implementation of temporary policies in response to the COVID-19 pandemic. Of 36 U.S. states having smoking restrictions as of October 2021, 20 have prohibited smoking in casinos and 30 in bars [1]. COVID-19 led to commercial casinos in New Jersey [2], Pennsylvania [3] and Michigan [4] and over 160 sovereign tribal casinos implementing temporary smokefree indoor air policies, starting debates on making them permanent. In November 2021, the Navajo Nation made all its casinos permanently smokefree as part of a larger clean indoor air law [5].

The years-long battle for smokefree laws in Louisiana helped lay the foundation for local ordinances there covering bars and casinos. The 2006 Louisiana Smoke-Free Air Act, passed with support from advocates who had sought to replace ineffective statewide smokefree laws since 2001, exempted bars and casinos [6]. The Coalition for a Tobacco-Free Louisiana (CTFLA), consisting of national and state health organizations, accepted the exemptions to avoid political resistance. Significantly, the law repealed state preemption of stronger local ordinances enacted with tobacco industry support. Since the 2006 law did not cover all workplaces and political inertia at the state level prevented comprehensive statewide smokefree legislation, health organizations pursued local smokefree ordinances starting in 2011 to extend smokefree policy coverage to bars and casinos. Louisiana’s nonprofit tobacco control program, Louisiana Campaign for Tobacco-Free Living (TFL), working with national, state and local organizations (including as a member of CTFLA), secured 30 local laws between 2011 and 2021 [7] despite resistance from the bar and gaming industries. The local smokefree ordinance battles in Louisiana provide insight into overcoming political opposition and securing smokefree laws for workplaces not yet protected by state laws.

To understand the success of campaigns pursuing comprehensive local smokefree ordinances covering bars and casinos in Louisiana, we analyzed the efforts of advocates and health organizations to pass local laws between 2011 and 2021. We found that health organizations overcame industry opposition to smokefree bars and casino interests with innovative campaigns expanding upon established smokefree organizing tactics [8,9,10,11,12] (extended and consistent media campaigns and news media engagement on secondhand smoke, messaging focusing on health and workplace protection, multilevel alliances, local organizing and countering industry claims) by integrating jazz musicians in messaging and campaign events to highlight how smokefree laws would protect employees as well as promote local culture. 

This paper also tests the applicability of the Policy Dystopia Model [13] (PDM) for predicting non-tobacco industry behavior against smoking restriction laws. The PDM is a framework that was developed to understand tobacco industry discursive and instrumental strategies against taxes and advertising restriction legislation. Discursive strategies outlined by the PDM focus on predicting secondary adverse social and economic outcomes of tobacco control legislation while instrumental strategies include coalition building, litigation, information management and policy interference to support tobacco industry positions. While the model was created by analyzing tobacco industry behavior against tax increases and advertising restrictions, it has been used to interpret tobacco industry opposition to smokefree laws in countries outside the United States [11,14]. The tobacco industry mobilized opposition nationally against smokefree policies in bars and casinos since the 1990s by arguing smokefree laws harmed those businesses in order to develop alliances with trade associations [15,16]. We compared casino industry behavior directed against smokefree ordinances in New Orleans and Baton Rouge to categories of arguments and strategies outlined by the PDM to determine if a non-tobacco industry would use tactics established by the model when opposing tobacco control policies. Louisiana’s experience shows that the PDM can be used to understand and anticipate gaming industry tactics against smokefree policies.

## 2. Materials and Methods

We performed a case study to understand health organization tactics and activities around passing local smokefree laws in Louisiana that combined information from the documentary record with key informant interviews. Snowball searches [17] were conducted for news on the Access World News Database, Google, and websites for the *Times-Picayune*/nola.com (New Orleans) and *The Advocate* (Baton Rouge) between 2000 and 2020. Search terms included “smoking,” “smoking restrictions,” and “ordinance,” followed by searches of key actors, organizations and locations. Key actors were those involved as campaign officials, operatives or representatives from business, government or advocacy organizations supporting or opposing smokefree ordinances. Key organizations were industry associations, businesses and health organizations that were involved in smokefree advocacy or policy campaigns as supporters or opponents, while locations were places where smokefree legislation was debated. News stories were read and cited if they added context or information on campaign activities, attitudes, messaging or strategies to local smokefree ordinance battles. 

Interviews were conducted with employees from 3 national and 4 state health organization representatives. Participants were approached if they served as health organization staff involved in local ordinance efforts, media messaging or were campaign officials in the New Orleans or Baton Rouge smokefree law campaigns in Louisiana. Interviews were performed under a protocol approved by the UCSF Committee on Human Research. We did not track who declined interviews, nor did we approach opponents of smokefree legislation for interviews since the focus of our research was on health organization strategies. Interview questions were unstructured and questions were developed organically based on information collected during research and prior interviews. Interviews were transcribed. TW and SAG have interacted with some of the interviewees at public health meetings. Two interviewees (Cynthia Hallett and Jennifer Cofer) serve on the external advisory committee for the UCSF Center for Tobacco Control Research and Education.

Our paper particularly analyzes smokefree battles in New Orleans and Baton Rouge. Both cities warranted focus because they are the largest population and tourism centers within Louisiana, they manifested the most intense efforts by casino entities to defeat smokefree coalitions and smokefree legislation, and represented the largest deployments of health organizations’ planning, coalition building and resources. New Orleans and Baton Rouge were two of three jurisdictions among the 30 towns and cities that prohibited smoking in bars and casinos in Louisiana that had operating casinos at the time the law was being debated.

We analyzed gaming industry behavior from smokefree ordinance campaigns in New Orleans and Baton Rouge to determine similarities and differences with tobacco industry tactics outlined by the PDM. We compared data to the PDM’s discursive and instrumental categories and subcategories. Arguments and activities that matched the PDM were placed in the corresponding category.

There are two reference lists for this paper. The references enclosed in square brackets and preceded with “S” refer to original source materials and appear in the Supplement File.

## 3. Results

### 3.1. Laying the Groundwork with a Focused Media Campaign

TFL launched a paid advertising and social media campaign “Let’s Be Totally Clear” in 2010 to advocate for employees and musicians’ rights to smokefree air [18]. The campaign directed viewers to TFL’s website with advocacy resources [19]. Rebranded as “Healthier Air For All” in 2012 according to one interviewee [20], the campaign generated capacity for ordinances.

### 3.2. Local Campaigns Build Support

TFL initially won comprehensive ordinances in localities without significant gaming industry presence, beginning in Alexandria in 2011 [21] after it responded favorably to TFL’s media campaign and grassroots education efforts according to one interviewee [22]. TFL organized smokefree events, provided promotional packages to bars and bingo halls and advertised on billboards and social media. One interviewee stated that TFL conducted air quality studies finding hazardous secondhand smoke (SHS) levels in local bars, prepared packets for lawmakers and recruited and trained speakers for hearings [22]. A law was passed, and TFL ran local ads to assist with its implementation [23].

Five additional municipalities adopted ordinances between 2012 and 2014 (Table 1), providing experience to pursue laws in New Orleans, Baton Rouge and elsewhere. While most locations that adopted comprehensive smokefree legislation covering bars and casinos did not host casinos such as New Orleans or Baton Rouge, the laws generated momentum and normalization for prohibiting smoking in places that had casinos.

TFL conducted research and ran its media campaign in New Orleans to generate support for an ordinance. It produced five studies on SHS and air quality, the economy and health relevant to New Orleans between 2011 and 2014 [26,27] using “Healthier Air for All and an affiliated statewide smokefree concert series held there in May 2014 [28].

### 3.3. Smokefree NOLA

In August 2014, CTFLA members formed Smokefree NOLA (the acronym for New Orleans) (Table 2). Four interviewees recalled that the coalition formed after New Orleans councilmember LaToya Cantrell told health organizations she was introducing a comprehensive ordinance [29,30]. Smokefree NOLA (SFNOLA) shared TFL’s statewide media campaign’s themes and branding [23,31].

**Figure 1:**
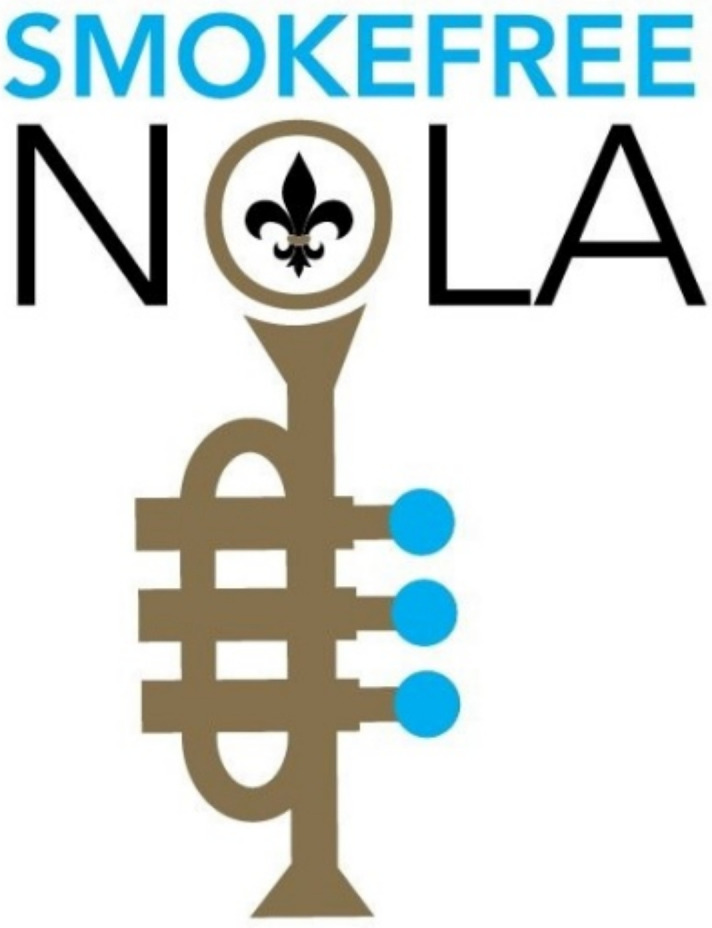
Smokefree NOLA Campaign Logo used the fleur-de-lis, a symbol of New Orleans’ French origins, and a trumpet, reflecting the city’s jazz legacy.

SFNOLA branding celebrated New Orleans’s culture and musical heritage while linking with Healthier Air For All [23,29,31,33] (Figure 1). One interviewee stated SFNOLA recruited musician spokespersons through the Louisiana Cultural Economy Foundation [34], which helped performers obtain economic assistance and healthcare [35]. TFL had partnered with the foundation since 2011 to hold smokefree music events [34]. Musicians wrote letters and attended hearings and campaign events [36]. 

SFNOLA held “Smoke-Free Week 2.0” in November 2014 to promote smoking restrictions [37] during the American Public Health Association’s (APHA) annual meeting (around 12,000 attendees [38]) in New Orleans [39]. The ordinance was introduced on the last day of Smoke-Free Week [37,40].

SFNOLA leveraged APHA’s meeting to press for legislation [29,30], with four interviewees recalling the coalition persuading APHA to declare it would not meet again in New Orleans unless the city adopted an ordinance meeting APHA’s smokefree meetings policy [41]. The American Heart Association, which held a large meeting (17,000 attendees [42]) in New Orleans every few years also threatened to avoid the city until a law was enacted [43]. 

SFNOLA organized promotions to engage the public. During Smoke-Free Week 2.0 it held a traditional New Orleans second line parade [44] that rallied for the ordinance at APHA’s meeting [45]. According to two interviewees, the coalition hosted smokefree events at LGBT and African-American bars, industry nights for service employees and placed announcements in churches [30]. It held a townhall [30,46], prayer breakfast and rally in January [46] and other events promoted on TFL’s Healthier Air for All website [36]. 

SFNOLA obtained USD 2.8 million in earned media by February 2015 [47] and funded advertising using partner contributions. 

### 3.4. Business Opposition

Gaming, bar and restaurant entities formed the Freedom to Choose Coalition to oppose the ordinance. One founder, Harrah’s [48], operated Louisiana’s only land-based casino. Other participants were the Louisiana Amusement and Music Operators Association, Louisiana Video Gaming Association, Louisiana Casino Association [49], French Quarter Business League, Louisiana Restaurant Association (LRA) and Louisiana Association of Wholesalers (LAW) [49]. LRA helped the tobacco industry resist statewide smokefree legislation in the 1980s and 1990s [6]. Altria and other tobacco companies sponsored LAW in 2015 [50] and its executive director worked for Philip Morris as a coordinator during the 1990s [51,52]. LAW’s director also had Altria (Philip Morris) as his lobbying firm’s client [53].

New Orleans’ vapers fought to exclude e-cigarettes from the ordinance [6], including speaking at the subcommittee hearing. According to two interviewees, local e-cigarette retailers formed the Louisiana Association of Electronic Cigarette Retailers (LAECR) to oppose the ordinance [30]. An out-of-state retailer [54] and the national Consumer Advocates for Smoke Free Alternatives Association [55] supplemented LAECR with action alerts encouraging e-cigarette advocates to attend town halls and contact lawmakers. 

### 3.5. Hearings

The New Orleans City Council considered the ordinance at a subcommittee hearing on 7 January [56] and a full hearing on 22 January [57].

The subcommittee started with a panel of health experts, a musician and a nightclub owner who testified on SHS’s dangers [56], countered economic harm claims and argued workers lacked choice regarding working in smoke. TFL provided councilmembers with briefs containing studies on air quality, SHS exposure, health effects, and the economic impact of smokefree laws [26,56].

At the hearings, health professionals, health organization representatives and residents supported the ordinance after being prepared by SFNOLA according to two interviewees [30]. They highlighted policy benefits, refuted economic harm claims [56,57], and argued smokefree laws protected employees and performers and their cultural contributions. They argued ventilation and smoking areas could not protect people from SHS and that e-cigarettes were underregulated and contained harmful components in aerosols.

Freedom to Choose argued the ordinance would harm businesses and employees, reduce tourism, lower tax revenue, drive customers to neighboring casinos and limit choice [56,57]. 

Harrah’s contracted with the state for its gaming license [58] and had a city lease [59]. The state contract prevented employment below 2455, while the lease required enhanced severance packages for employees terminated below 2550 [59]. Harrah’s claimed resulting revenue losses justified reducing employment required by its contract [56], a number it wanted lowered since 2007 [60].

Vaping proponents tried excluding e-cigarettes from the ordinance, arguing e-cigarettes are healthier than cigarettes and aid cessation [56]. They questioned research [56] and referenced statements from public health officials supporting legal access to e-cigarettes or their harm reduction potential including FDA Center for Tobacco Products Director Mitch Zeller and UK Royal College of Physicians Tobacco Advisory Group Chair John Britton [56]. LAECR referenced a World Health Organization letter advocating against prohibiting e-cigarettes [56]. (We could not locate any such letter.) LAECR accused the Council of succumbing to threats of losing conferences [57].

The Council unanimously adopted the ordinance on 22 January 2015 [57], effective 22 April [61]. 

### 3.6. Health Organizations Support Implementation despite Industry Resistance

SFNOLA members assisted with implementation. According to two interviewees, Americans for Nonsmokers’ Rights (ANR), a national organization, organized a meeting between the New Orleans Health Department and southern health officials to learn implementation strategies [29]. Four interviewees recalled that ANR helped fund the department’s implementation website and toolkits [29,30,36]. SFNOLA provided promotional materials to casinos and bars, advertised and sent education teams to local events.

Harrah’s resisted the ordinance, announcing in March 2015 that it sought exemptions to its contract because of expected revenue declines [62]. It offered to create smoking sections [63] and offered cessation services and smoking education to employees and customers to be exempted [63]. Harrah’s claimed the law threatened its lease payments and USD 3.6 million annually to New Orleans [63]. Harrah’s claimed it could renegotiate its lease if the city refused, which city officials rejected [62]. 

Harrah’s and 54 other bars, restaurants and strip clubs filed a class action lawsuit against the ordinance, claiming procedural errors and that the legislation was vague [64]. The court dismissed the case [65]. 

Harrah’s partnered with a state senator to introduce legislation allowing renegotiation of its state contract [60,66], including reducing Harrah’s employment requirement [67]. The Senate Judiciary Committee delayed consideration after a member found New Orleans officials were unaware of the attempted employment reduction [67]. New Orleans’ officials opposed the bill; the Committee rejected it [60]. 

The State Legislature’s Joint Budget Committee twice refused to renew the Louisiana Gaming Control Board’s contract with New Orleans for hosting Harrah’s, slowing USD 3.6 million [68], as threatened by Harrah’s [63]. Altria and Harrah’s shared lobbyists supported Harrah’s request with the committee [68], but the contract was renewed without change. 

Health organizations did not join this debate.

Harrah’s complied with New Orleans’ ordinance upon effect in April 2015 [69], but blamed it for revenue declines in May, June and August 2015 compared to the prior year; Harrah’s’ profits increased in July and September [70]. 

Health organizations contested Harrah’s economic assertions. TFL released a study in July 2015 finding Harrah’s was experiencing a 10-year revenue decline because of unrelated factors [71]. Harrah’s failed to resist the law, eventually building outdoor smoking courtyards for gaming [72].

A June 2015 air quality study found that hazardous indoor air quality improved to safe levels [73].

### 3.7. Forming Another Coalition for Baton Rouge in 2016

Louisiana’s state capitol, Baton Rouge, and its parish, East Baton Rouge, have a consolidated government [74]. Health organizations, many involved in CTFLA and SFNOLA, formed the Smoke-Free East Baton Rouge Coalition (SFEBR; Table 2) to pursue an ordinance.

A community meeting launched the campaign in January 2016 [75]. A SFEBR representative spoke to the local Rotary about protecting employees from SHS [76] and SFEBR hosted smokefree events, including a happy hour, karaoke night and music performance [77]. The American Association of Retired Persons Louisiana Chapter, Miss Louisiana, Baton Rouge musicians [78] and local hospitals and medical groups endorsed the coalition [79,80]. 

SFEBR spent approximately USD 300,000 on advertising in its first six months for radio, billboards, social media and television [81]. The coalition disseminated SHS facts, articles on smokefree policies, casino employee testimonials and action alerts via social media [77,82]. 

### 3.8. Opposition

Gaming industry resistance started in March 2016. L’Auberge Casino owner, Pinnacle Entertainment, claimed the law would reduce income and tax revenue, referencing fallen profits after smoking bans [83,84]. In April, as hearings neared, Baton Rouge’s three casinos claimed they expected the ordinance to inflict economic harm, reduce tax revenues, disadvantage them with smoking venues and potentially reduce their purchases from local vendors [85]. They argued that SHS was not problematic in employee surveys and the ordinance would harm workers by lowering business and tips [85]. 

### 3.9. Hearings

East Baton Rouge’s Metro-Parish Council considered the ordinance in April 2016 [86]. Health organization representatives, doctors, nurses, entertainers, faith leaders and casino employees asserted the ordinance protected personal rights and health and did not harm businesses, particularly casinos.

Gaming officials and workers testified that smokefree laws harmed casino and tax revenue and reduced purchases from local vendors [86]. They argued that working in SHS was a choice and asserted that ventilation made the ordinance unnecessary, a common tobacco industry argument [16,86]; ventilation cannot prevent harmful SHS exposure [87]. L’Auberge employees and officials claimed workers desiring smokefree areas were accommodated [86]. The Council voted six to six, defeating the ordinance [86].

### 3.10. Pursuing Ordinances throughout Louisiana

Municipalities, many with TFL’s assistance, won ordinances in seven Louisiana communities between 2015 and 2017 (Table 1) to build capacity [6]. 

### 3.11. The Second Baton Rouge Campaign in 2017

SFEBR announced in May 2017 that it would pursue another ordinance [88]. and in June, 7 out of 12 East Baton Rouge Metro-Councilmembers co-sponsored legislation [89].

The coalition disseminated information, secured endorsements and held events to support the ordinance. It released a poll showing local women supported a law by 79% and college educated women supported the law by 69% [90]. SFEBR organized a smokefree bar night, happy hours, music performances, a comedy night and a dance night, and circulated flyers, informational videos, tobacco health statistics, news, blog posts and lawmakers’ contact information [77,82]. It also partnered with Miss Black Louisiana U.S. Ambassador LeighAnna Kingvalsky for promotional efforts [77,91], Three ordinance sponsors participated in a local radio show [92,93,94]. SFEBR secured letters in the Baton Rouge *Advocate* [91,95] and released an air quality study on local casinos and bars finding unhealthy air conditions [96].

### 3.12. Hearings

The Baton Rouge Metro-Parish Council considered the second ordinance in June [97] and August 2017 [98]. 

Coalition members, health representatives, city employees, performers, bar industry members and locals supported the ordinance [97,98]. They highlighted SHS’s harms and costs, asserted the right to smokefree workplaces, discussed local bars and casinos’ poor air quality and how smokefree policies improved public health. Proponents countered claims that restrictions harm income and that ventilation systems “solve” SHS. They reported 20 states and various localities prohibited smoking in casinos. 

Opponents argued smokefree laws cost jobs, profits, employee income and tax revenue. They asserted customers and employees chose to frequent casinos [97,98] while claiming workers wanting smokefree environments were accommodated, that most employees did not work in smoking areas, and that ventilation protected people.

Baton Rouge lawmakers approved the ordinance with an effective date of 1 June 2018 [98]. 

SFEBR supported implementation by promoting the law and SHS’s harmfulness on social media, sponsoring events [77,99], producing implementation toolkits [100] and educating Baton Rouge police about the ordinance [101]. An air quality survey conducted a month after implementation found a 98.8% improvement in air quality in places that previously allowed smoking [102].

## 4. Discussion 

Louisiana illustrates how health organizations can shift to local campaigns to secure ordinances covering bars and casinos when state progress is blocked. Local governments are more responsive to constituents where the tobacco industry [103] and other sectors [104] have less influence on policymaking. After state smoking restrictions stagnated following 2006, Louisiana health organizations pursued local comprehensive ordinances covering casinos and bars, enabled by the 2006 repeal of preemption. Louisiana organizations sustained their partnerships after 2006, allowing deployment of an existing coalition network that facilitated cooperation [105] to pass comprehensive local ordinances over business resistance. Starting in 2011, TFL secured ordinances using policy campaigns supported by its “Let’s Be Totally Clear/Healthier Air for All” media initiative. Efforts to pass local smokefree ordinances in Louisiana serve as a model for passing comprehensive protections in states that currently lack statewide smokefree protections for bars and casinos because of political resistance at the state legislature, as long as they are not preempted by state law.

### 4.1. A Theoretical Framework: The Policy Dystopia Model

The PDM [13] identifies two forms of resistance, discursive and instrumental. Discursive arguments seek to cast regulation as economically harmful to the economy or society, as criminalizing or crime generating, are unbeneficial to public health, regressive, ineffective, beneficial to undeserving interests and a form of government overreach as well as harmful to business interests and employees. Instrumental tactics include funneling information to the public that benefits the industry’s image and position, hides its role in information sharing, weakens public health organizations’ claims and standing and portrays the regulation as ineffective. Other instrumental strategies include recruiting or manufacturing allies, breaking public health alliances, suing and directly interfering in the policy process.

Louisiana indicates the gaming industry, which has opposed smoking restrictions in partnership with the tobacco industry [16], deployed PDM tactics (Table 3). In New Orleans and Baton Rouge, they claimed the ordinances would inflict economic and tax harm while denying their effectiveness. Harrah’s formed a coalition, participated in a class action lawsuit and interfered in legislative and contractual processes in attempts to block the New Orleans ordinance. None of the casinos in New Orleans or Baton Rouge used PDM strategies such as discursive arguments that smokefree policies benefited undeserving groups or inadvertently harmed public health, nor did they engage in illicit trade. Gaming industry strategies indicate that, similar to the tobacco industry, they seek external institutional lanes to failing policy venues where they have more influence. Louisiana shows that the PDM can be used to understand industry opposition to smokefree laws inside and outside the Unites States [11,14], not just opposition to advertising restrictions and taxes, which were used to develop the PDM.

### 4.2. Health Coalition Effectiveness in Countering Industry Political Influence

TFL’s ongoing “Healthier Air for All” media campaign on the effects of SHS [106] and social justice themes [107] facilitated ordinance campaigns across Louisiana. TFL supplemented these general messages with SHS-impacted musicians and other performers to equate smokefree environments to preserving local culture, while also messaging the need for workplace protections. Focusing on policy solutions through those impacted by a policy change reoriented the media narrative from victim responsibility to a public issue [108]. Cobranded local campaigns tied smoking restrictions to a statewide movement, using norm changes to enable victories. 

In Louisiana, as in other successful campaigns inside [8,9] and outside [10,11] the United States, joining with health organizations at higher political levels provided resources and expertise to combat well-financed and coordinated industry resistance. Louisiana’s framing around musicians and performers, workers and customers in an adult rights and cultural context, as opposed to youth as was conducted in Duluth Minnesota [8], facilitated the passage of stronger ordinances than in Duluth, where focusing on youth led to weak restrictions allowing smoking sections in restaurants, exemptions for some restaurants and smoking in bars. 

SFNOLA and SFEBR’s aggressive media engagement via events and outreach as well as advertisements countered casino industry claims of economic harm and highlighted the need to protect workers. This experience is consistent with Mexico City, where a coalition of international and state organizations obtained and defended a smokefree law [10]. The Mexico City campaign, similar to the Louisiana campaigns, had dedicated legislative champions and promoted the protective and beneficial qualities of smokefree laws. Comparing local campaigns in Louisiana, El Paso [9] and Mexico City [10] to Duluth [8] indicates that consistent media outreach, messaging around worker and public protection, collaborating with strong legislative champions, countering opponents’ economic arguments and relying on support from larger health organizations are essential to campaigns seeking comprehensive legislation. 

### 4.3. Limitations

This paper relies on interviews and testimony from people involved with government and health organizations. Our findings from interviews may be influenced by personal bias, as not all health organization participants were interviewed, or by recall bias since years have passed from the events covered. Interviews were conducted between 2014 and 2017, so subjects could have forgotten or recalled details incorrectly. We were unable to obtain detailed funding information for local campaigns in Louisiana, preventing analysis of the financial asymmetry between smokefree proponents and opponents. We relied on news coverage and hearings to analyze bar and gaming industry behavior, because in the past, representatives from these groups consistently declined to be interviewed. We may have missed clandestine tobacco industry involvement. 

## 5. Conclusions

Louisiana health organizations secured 30 local smokefree laws covering casinos and bars, including in New Orleans and Baton Rouge, between 2011 and 2021. Louisiana’s experience indicates that effective established strategies for enacting smokefree laws (a sustained media campaign, local organizing, polling and countering industry claims) can be combined with an emphasis on worker protections and local culture to mount successful campaigns to enact smokefree laws.

## Figures and Tables

**Table 1:** Localities in Louisiana with 100% Smokefree Laws including Casinos [7,24,25].

Year	Number of Cities that Adopted Smokefree Law Covering Casinos	Number of Cities with Casinos	City
2012	2	0	Alexandria
			Woodworth
2014	4	0	Cheneyville
			Monroe
			Ouachita Parish
			West Monroe
2015	3	1	Hammond
			New Orleans *
			Abbeville
2016	1	0	Bogalusa
2017	5	1	Glenmora
			Lafayette Parish
			Town of Lecompte
			Baton Rouge/East Baton Rouge Parish *
			Colfax
2018	2	0	Roseland
			Village of McNary
2019	12	0	Fenton
			Boyce
			Cullen
			Ruston
			Pineville
			Ponchatoula
			Haynesville
			Natchez
			Reeves
			Oak Grove
			Athens
			Angie
2020	1	1	Shreveport *

* Casino operating when ordinance was being considered.

**Table 2:** Composition of the Smokefree NOLA and Smoke-Free East Baton Rouge Coalitions [27,29,30,32].

	Smokefree NOLA	Smoke-Free East Baton Rogue
National Health Voluntaries	American Cancer Society Cancer Action Network	American Cancer Society Cancer Action Network
	American Heart Association	Red Team
	American Lung Association	American Lung Association
	Americans for Nonsmokers’ Rights	Americans for Nonsmokers’ Rights Foundation
	Campaign for Tobacco-Free Kids	Campaign for Tobacco-Free Kids
	March of Dimes	March of Dimes
Louisiana Organizations	Tobacco-Free Living/Louisiana Public Health Institute	Tobacco-Free Living/Louisiana Public Health Institute
	Louisiana Comprehensive Cancer Control Partnership	Musicians for a Smoke-Free Louisiana
	Oschner Health Systems	National Association of Social Workers Louisiana Chapter
	Louisiana Cultural Economy Foundation	Louisiana Budget Project
	LSU Tobacco Control Initiative	--
	Smoking Cessation Trust	--
Local Organizations	Communities of Color Network	SEIU Local 21 LA
	Fresh Campus Campaign	--

**Table 3:** Comparison of the Policy Dystopia Model and use of its Tobacco Industry Discursive and Instrumental Strategies by the Gaming Industry in Major Louisiana Policy Battles [6,13].

	Themes	New Orleans	Baton Rouge
Discursive	Unanticipated Costs to Economy and Society	- Harms casino revenue - Harms tax revenue - Harms tourism - Reduces funding for law enforcement	- Harms casino revenue - Harms tax revenue - Harms local vendors dependent on casinos - Business choice
	Unintended Benefits to Undeserving Groups	Not used	Not used
	Unintended Costs to Public Health	Not used	Not used
	Denial of Intended Public Health Benefits	Not used	- Ventilation systems already clean air - Majority of establishments are nonsmoking - Casino employees are accommodated - Customers can attend casinos that permit no smoking if they desire - Not a workplace that all members of the public must frequent (grocery stores for example) - Secondhand smoke studies are unreliable and lack quality
	Expected Industry Costs *	- Casino employees will lose income and work hours - Casino employees will lose jobs	- Casino employees will lose income - Casino employees will lose jobs
Instrumental	Coalition Management	- Harrah’s helped form the Freedom to Choose Coalition.	Not used
	Information Management	- Repeatedly blamed smokefree ordinance for revenue declines	Not used
	Direct Involvement and Influence in Policy	- Threats by Harrah’s to renegotiate contract with New Orleans and the State - Legislation to renegotiate Harrah’s contract and reduce employment - Louisiana gaming board delays casino contract approval	Not used
	Litigation	Harrah’s and its allies filed a lawsuit to repeal New Orleans’ smokefree law	Not used
	Illicit Trade	Not used	Not used

* The Policy Dystopia Model was built using the tobacco industry’s behavior for its marketing and tax policy efforts, and this category was specifically labeled for the tobacco industry. We modified the label to refer to the casino industry.

## Data Availability

Interviews have been deposited in the UCSF Tobacco Control Archive, maintained by Archives and Special Collections at the UCSF Library. All other materials are publicly available at the cited sources.
